# Identifying Single-Cell Expression Quantitative Trait Loci Using a Bootstrap Penalized Hurdle Model

**DOI:** 10.3390/genes17060625

**Published:** 2026-05-29

**Authors:** Dongyuan Wu, Susmita Datta

**Affiliations:** Department of Biostatistics, University of Florida, Gainesville, FL 32611, USA

**Keywords:** eQTL analysis, hurdle model, single-cell, bootstrap, penalized regression

## Abstract

Background: Expression quantitative trait loci (eQTL) analysis links genetic variants to gene expression levels, helping to uncover how genetic variation contributes to gene regulation. While traditional eQTL analyses rely on bulk RNA-seq data, recent advances in single-cell RNA sequencing (scRNA-seq) have made it possible to detect cell-type-specific eQTLs. However, the inherent sparsity and heterogeneity of scRNA-seq data present major challenges for standard modeling approaches. Methods: In this paper, we propose a novel statistical framework, Bootstrap Penalized Hurdle regression model (BPHurdle), designed specifically for scRNA-seq data. BPHurdle employs a hurdle modeling framework, where a logistic component accounts for the excess zeros in single-cell expression data, and a Poisson component jointly evaluates the effects of multiple SNPs on positive gene expression levels. Results: Through simulation studies, we show that BPHurdle achieves high accuracy and robustness in identifying regulatory variants. We further demonstrate its utility on a real dataset through a case study focusing on a subset of differentially expressed genes, where it successfully identifies reliable cell-type-specific eQTLs. Conclusions: Overall, BPHurdle offers an advanced and flexible approach for single-cell eQTL mapping, providing deeper insight into the genetic regulation of gene expression at cellular resolution.

## 1. Introduction

In recent decades, genome-wide association studies (GWAS) have significantly enhanced our understanding of the genetic basis of complex traits and diseases [1]. GWAS aims to uncover connections between genotypes and phenotypes by examining discrepancies in allele frequencies of genetic variants, such as single-nucleotide polymorphisms (SNPs), across individuals with varying phenotypes [2]. However, further comprehending the contribution of disease-associated genetic variants identified through GWAS to pathogenesis remains challenging [3]. We know that gene expression, or transcription, serves as a pivotal intermediary step in the molecular mechanism chain linking genotype to phenotype [4,5]. Consequently, investigating the influence of genetic variants on regulating gene expression levels becomes imperative; this field is known as expression quantitative trait loci (eQTL) analysis. Notably, eQTL analysis has a similar form to GWAS, utilizing gene expression levels as phenotypic traits to discern their relationship with genotypes. This approach has emerged as a potent tool for elucidating the regulatory mechanisms of genetic variants that govern gene expression variation, thereby offering valuable biological insights [6,7].

Many eQTL studies have traditionally relied on bulk RNA-seq data, which captures average gene expression levels across all cells within an individual. For example, MatrixEQTL [8] stands as the predominant software in bulk eQTL studies. It initially selects gene–SNP pairs based on a threshold of test statistics, such as the absolute value of the sample correlation coefficient. Subsequently, it performs linear regression and tests for significance of each selected gene–SNP pair. This method has been valuable for inferring potential general regulatory effects of SNPs on genes across various cell types. However, emerging evidence suggests that eQTL effects may vary between different cell types or states [9,10]. In other words, eQTLs can exhibit cell-type-specific characteristics. Although researchers have dedicated efforts to developing methods for deconvoluting cell type proportions and mapping eQTLs from bulk RNA-seq data [11,12,13], a more precise technology capable of directly providing gene expression values at the cell level would be preferable for identifying cell-type-specific eQTLs. scRNA-seq technologies offer us the opportunity to obtain gene expression information at single-cell resolution, which can naturally be segregated into different cell types and aid in mapping cell-type-specific eQTLs [14]. However, since genotype information is at the individual level, it is necessary to align genotype information (individual level) with transcription information (single-cell level).

One solution is to reconstruct pseudo-bulk gene expression levels across all cells for a specific cell type to individual level from the scRNA-seq data, and then perform traditional eQTL analysis [15]. We refer to these as pseudo-bulk models. Alternatively, another approach is to retain single-cell resolution by extending genotype information to each cell of the individual for individual cell modeling, termed single-cell models. This strategy preserves more information on cell heterogeneity and may yield more precise mapping of regulatory genetic variants on genes. It is important to note that single-cell models are not suitable for using existing methods designed for traditional eQTL analysis on bulk RNA-seq data, due to their differing properties and distributions.

In recent years, several single-cell models have been proposed. For example, Hu et al. [16] proposed a zero-inflated negative binomial regression model to account for the high probability of gene expression being zeros in single-cell data. Other models attempt to use simpler distribution assumptions, such as normal distribution or Poisson distribution, but incorporate more complex model structures, including multiple random effects and interaction terms, to account for biological aspects [17,18,19,20,21]. However, current methods only consider one gene–SNP pair at a time using univariate regression models. While this simplifies the model and provides marginal regulatory effects, it may introduce bias in the results, as multiple SNPs may jointly influence gene expression levels; such limitations of univariate analyses have been discussed in previous studies [22,23]. Sparse and penalized regression frameworks have been explored in eQTL mapping to jointly model the effects of multiple genetic variants, addressing limitations of traditional univariate approaches that test each SNP independently [24,25]. However, these approaches have primarily focused on bulk RNA-seq data and standard regression frameworks, and do not account for zero inflation or the unique sparsity structure of scRNA-seq data. Together, these gaps motivate the development of models that can simultaneously account for sparsity in scRNA-seq data and jointly evaluate the effects of multiple SNPs.

In this paper, we propose a novel single-cell statistical model for mapping cell-type-specific eQTLs, designed to accommodate the unique characteristics of scRNA-seq data, which often exhibits sparsity with a high proportion of zeros and skewed distribution of expression patterns [20,26,27,28,29]. Specifically, we develop a bootstrap penalized hurdle regression model (BPHurdle) with a Poisson distribution. This model enables the simultaneous exploration of the association between a specific gene and a series of SNPs within eQTL analysis. We validate that BPHurdle can accurately discover the regulatory effects of genetic variants for each gene without compromising much specificity in simulated studies compared to other algorithm frameworks. Furthermore, we show that BPHurdle can detect reliable cell-type-specific eQTLs in real data applications.

The rest of this manuscript is organized as follows. Section 2 provides an overview of the proposed BPHurdle model, including detailed model structure and inference. In Section 3, we conduct two simulation studies using data generated from different distributions to demonstrate the accuracy, reliability, and robustness of the proposed model. In Section 4, we present a case study to showcase the usability of the proposed model. Finally, in Section 5, we summarize our conclusions and provide a discussion.

## 2. Materials and Methods

**Figure 1:**
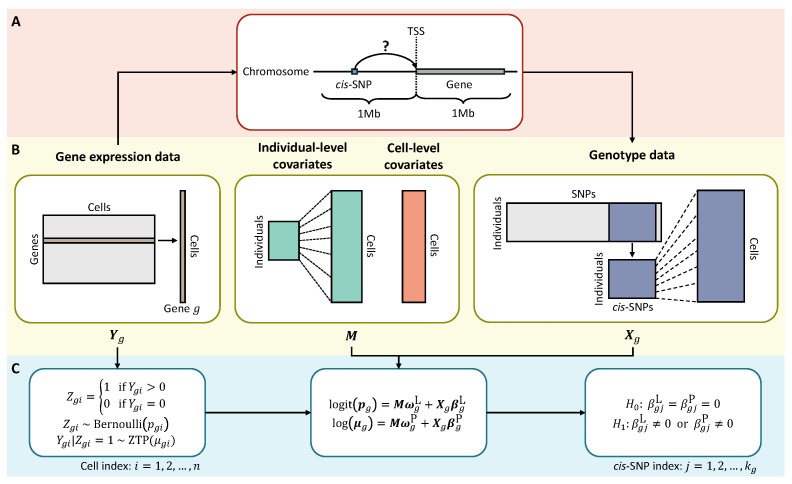
Overview of the BPHurdle framework. (**A**) Illustration of the study objective, where the goal is to identify *cis*-eQTLs for genes using single-cell data. (**B**) Required inputs to the BPHurdle model, including single-cell gene expression data ($Y_{g}$), corresponding proximal genotype data (M), and additional covariates such as confounding factors and population structure ($X_{g}$). (**C**) Workflow of the BPHurdle model. The framework consists of two components: a logistic component modeling the probability of zero versus non-zero expression ($p_{g}$), and a zero-truncated Poisson component modeling positive expression values ($\mu_{g}$). These components are integrated within a penalized regression framework, followed by bootstrap-based hypothesis testing to identify significant eQTLs.

The framework of our proposed approach is summarized in Figure 1. eQTL studies can be categorized into two types: those with proximal effects (*cis*-eQTLs) and those with distant effects (*trans*-eQTLs). *Cis*-eQTLs are genetic variants located near the affected gene, typically on the same chromosome and within 1 Mb of the gene’s transcription start site (TSS). In contrast, *trans*-eQTLs are genetic variants located far from the affected gene, either more than 1 Mb away or on a different chromosome [30,31]. For clarity, we will focus exclusively on mapping *cis*-eQTLs and treat it as our goal within the BPHurdle framework (Figure 1A).

The BPHurdle framework requires a gene expression matrix from scRNA-seq data to obtain cell-level gene expression counts, as well as a genotype dosage matrix, with values of 0, 1, or 2 representing the number of alternative alleles of a specific SNP for each individual. For a given gene, we extract its *cis*-SNPs from the genotype dosage matrix and expand them to the cell level. Additionally, we can include covariates that are confounders or related to population structure. After fitting the hurdle Poisson regression model, the eQTL effects can be inferred from the regression coefficients. If a SNP affects a gene, the SNP is called an eSNP and the affected gene is called an eGene.

### 2.1. Hurdle Model Structure

To facilitate the mapping of eQTL for a specific gene, we propose a hurdle model structure designed to address both the excess zeros and overdispersion issues inherent in the positive counts observed in single-cell data. Let us define $Y_{gi}$ as the expression count of gene *g* (g=1,2,…,G) in cell *i* (i=1,2,…,n), with $Z_{gi}$ indicating whether gene *g* is expressed in cell *i*. This can be represented as follows:

$$Z_{gi}=\left\{\begin{array}{cc} 1, & \mathrm{if}\;Y_{gi}>0,\\ 0, & \mathrm{if}\;Y_{gi}=0. \end{array}\right.$$

We assume the indicator variable $Z_{gi}$ following a Bernoulli distribution $\mathrm{Bernoulli}(p_{gi})$, and the positive counts $Y_{gi}|Z_{gi}=1$ as conditionally following a zero-truncated Poisson distribution $\mathrm{ZTP}(\mu_{gi})$. Consequently, the probability mass functions of $Z_{gi}$ and $Y_{gi}|Z_{gi}=1$ are defined as:

(1) $$\mathrm{Pr}\left(Z_{gi}=z\right)=p_{gi}^{z}{\left(1-p_{gi}\right)}^{1-z},z=0,1,$$

(2) $$\mathrm{Pr}\left(Y_{gi}=y|Z_{gi}=1\right)=\frac{\mu_{gi}^{y}\exp\left(-\mu_{gi}\right)}{y!(1-\exp\left(-\mu_{gi}\right))},y=1,2,\ldots ,$$

where $p_{gi}\in \left(0,1\right)$ represents the probability of gene *g* being expressed in cell *i*, and $\mu_{gi}>0$ denotes the mean of non-zero expression count.

Utilizing Equations (1) and (2), we can independently fit the hurdle Poisson regression model as follows:

(3) $$\mathrm{logit}\left(p_{g}\right)=M\omega_{g}^{L}+X_{g}\beta_{g}^{L},$$

(4) $$\log\left(\mu_{g}\right)=M\omega_{g}^{P}+X_{g}\beta_{g}^{P}.$$

Here, $X_{g}$ represents a ($n\times k_{g}$) matrix of genotype information for $k_{g}$ *cis*-SNPs of the specific gene *g* across *n* cells, and the vectors $\beta_{g}^{L}={\left(\beta_{g1}^{L},\beta_{g2}^{L},\ldots ,\beta_{gk_{g}}^{L}\right)}^{T}$ and $\beta_{g}^{P}={\left(\beta_{g1}^{P},\beta_{g2}^{P},\ldots ,\beta_{gk_{g}}^{P}\right)}^{T}$ include the corresponding regression coefficients for the $k_{g}$ *cis*-SNPs, serving as our parameters of interest. On the other hand, M is a (n×(q+1)) matrix comprising a column of all 1’s followed by any additional *q* covariates considered in the model, such as age, gender, and population structures. The vectors $\omega_{g}^{L}={\left(\omega_{g0}^{L},\omega_{g1}^{L},\ldots ,\omega_{gq}^{L}\right)}^{T}$ and $\omega_{g}^{P}={\left(\omega_{g0}^{P},\omega_{g1}^{P},\ldots ,\omega_{gq}^{P}\right)}^{T}$ correspond to the model intercept and the regression coefficients for the *q* covariates. The superscripts L and P distinguish the regression coefficients $\beta_{g}$ and $\omega_{g}$, corresponding to the logistic model for $Z_{g}$ and the zero-truncated Poisson model for $Y_{g}|Z_{g}=1$, respectively.

Based on Equations (1)–(4), the corresponding log-likelihood function is

(5) $$l\left(\beta_{g}^{L},\beta_{g}^{P},\omega_{g}^{L},\omega_{g}^{P}\right)=l_{1}\left(\beta_{g}^{L},\omega_{g}^{L}\right)+l_{2}\left(\beta_{g}^{P},\omega_{g}^{P}\right),$$

where $l_{1}\left(\beta_{g}^{L},\omega_{g}^{L}\right)$ and $l_{2}\left(\beta_{g}^{P},\omega_{g}^{P}\right)$ are the log-likelihood functions of the logistic regression model and the zero-truncated Poisson regression model, separately. The detailed derivation is provided in Appendix B.

### 2.2. Penalization

While our primary focus remains on the *cis*-eQTL analysis, it is essential to recognize that many SNPs proximal to a specific gene may not necessarily influence its regulatory mechanisms. Therefore, the critical task is to identify significant SNPs that genuinely impact gene expression amidst the surrounding noise within the multiple regression model. To achieve this, we will employ the elastic net penalization [32] on the regression coefficients of SNPs, i.e., $\beta_{g}^{L}$ and $\beta_{g}^{P}$, within the hurdle Poisson model (Equations (3) and (4)) for variable selection. Note that no shrinkage is applied to the coefficients for other covariates ($\omega_{g}^{L}$ and $\omega_{g}^{P}$) to ensure they are always included in the model.

From Equation (5), we can decompose the log-likelihood function into two separate parts: $l_{1}\left(\beta_{g}^{L},\omega_{g}^{L}\right)$ and $l_{2}\left(\beta_{g}^{P},\omega_{g}^{P}\right)$. Consequently, the penalties can also be applied to these two parts separately, and the estimates can be defined as follows:

$$\begin{array}{cc} ({\hat{\beta }}_{g}^{L},{\hat{\omega }}_{g}^{L})= & \arg\min_{\beta_{g}^{L},\omega_{g}^{L}}\left\{-\frac{1}{n}l_{1}\left(\beta_{g}^{L},\omega_{g}^{L}\right)+\lambda_{1}\left[\frac{1-\eta }{2}\left({∥\beta_{g}^{L}∥}_{2}^{2}\right)+\eta \left({∥\beta_{g}^{L}∥}_{1}\right)\right]\right\}, \end{array}$$

$$\begin{array}{cc} ({\hat{\beta }}_{g}^{P},{\hat{\omega }}_{g}^{P})= & \arg\min_{\beta_{g}^{P},\omega_{g}^{P}}\left\{-\frac{1}{n^{\prime }}l_{2}\left(\beta_{g}^{P},\omega_{g}^{P}\right)+\lambda_{2}\left[\frac{1-\eta }{2}\left({∥\beta_{g}^{P}∥}_{2}^{2}\right)+\eta \left({∥\beta_{g}^{P}∥}_{1}\right)\right]\right\}, \end{array}$$

where $n^{\prime }=\sum_{i=1}^{n}I\left(y_{gi}>0\right)$, and η∈[0,1] is a predefined parameter that can control the elastic net penalty. As η increases, the penalty strength increases. Specifically, η=1 represents the default lasso penalty, whereas η=0 corresponds to the ridge penalty. Additionally, $\lambda_{1}$ and $\lambda_{2}$ serve as tuning parameters that control the overall strength of the penalty [33,34]. We use 5-fold cross-validation to select the optimal values of $\lambda_{1}$ and $\lambda_{2}$ separately for the logistic and zero-truncated Poisson components, with each component optimized based on its own deviance. This allows each component to be tuned according to its own likelihood structure.

### 2.3. Model Inference

After selecting the optimal values of $\lambda_{1}$ and $\lambda_{2}$, we re-run the model on the complete dataset for inference. Typically, in eQTL studies, the focus of inference is to determine whether genes are significantly affected by certain SNPs. Within our modeling framework, this is governed by the pair of regression coefficients $\beta_{gj}^{L}$ and $\beta_{gj}^{P}$, where $j=1,2,\ldots ,k_{g}$ denotes the *j*-th *cis*-SNP for gene *g*. A gene is considered affected by the *j*-th *cis*-SNP if at least one of these two parameters, $\beta_{gj}^{L}$ and $\beta_{gj}^{P}$, is non-zero. Thus, we need to test $H_{0}:\beta_{gj}^{L}=\beta_{gj}^{P}=0$ against the alternative hypothesis.

In practice, inferring from the penalized regression model poses challenges. Therefore, we employ bootstrapping to construct bootstrap hypothesis testing for each parameter of interest and assess its significance. Suppose we sample the original data with replacement *B* times. We then obtain sample estimates ${\hat{\beta }}_{gj,b}^{L}$ and ${\hat{\beta }}_{gj,b}^{P}$ for the *b*-th resampling iteration, where b=1,2,…,B. In hypothesis testing, we compare the observed estimates with the null values (i.e., zeros). Thus, for the bootstrap test, we first need to construct a distribution under the null hypothesis $H_{0}:\beta_{gj}^{L}=\beta_{gj}^{P}=0$. To achieve this, we shift and center the bootstrapped samples around 0 to emulate the null distribution. Thus, the bootstrap *p*-values can be obtained by

$$p_{gj}^{L}=\frac{1}{B}\sum_{b=1}^{B}I\left(\left|{\hat{\beta }}_{gj,b}^{L}-\frac{1}{B}\sum_{b=1}^{B}{\hat{\beta }}_{gj,b}^{L}\right|\geq \left|{\hat{\beta }}_{gj}^{L}\right|\right),$$

$$p_{gj}^{P}=\frac{1}{B}\sum_{b=1}^{B}I\left(\left|{\hat{\beta }}_{gj,b}^{P}-\frac{1}{B}\sum_{b=1}^{B}{\hat{\beta }}_{gj,b}^{P}\right|\geq \left|{\hat{\beta }}_{gj}^{P}\right|\right),$$

where ${\hat{\beta }}_{gj}^{L}$ and ${\hat{\beta }}_{gj}^{P}$ are the estimates of coefficients $\beta_{gj}^{L}$ and $\beta_{gj}^{P}$ from original samples.

The *p*-values $p_{gj}^{L}$ and $p_{gj}^{P}$ are then adjusted for multiple testing cross $j=1,2,\ldots ,k_{g}$ using the false discovery rate (FDR) adjustment [35]. If either of the adjusted *p*-values is less than the defined significance level (usually 0.05), we reject the null hypothesis $H_{0}$ and conclude that gene *g* is affected by the *j*-th *cis*-SNP.

## 3. Simulation Study

We validated our modeling strategy and assessed its performance through extensive simulation studies. These studies utilized empirically observed genotype data to generate synthetic data. Initially, we randomly sampled 50 individuals from phase 3 of the 1000 Genomes Project [36], including 25 males and 25 females. We then randomly selected three genes from chromosome 2, which encompasses 1354 genes, and another three genes from chromosome 16, which encompasses 991 genes. The gene information used in the simulations is detailed in Table 1. Using the selected genes, we obtained the corresponding genotype information (i.e., *cis*-SNPs) from phase 3 of the 1000 Genomes Project [36] for the 50 selected individuals. Assuming each individual possessed 100 cells, we expanded the *cis*-SNP matrix from the individual level (50 rows) to the single-cell level (5000 rows). We then generated gene expression values using two different methods: our proposed hurdle Poisson model and the zero-inflated negative binomial model. Both methods mimic the properties of single-cell data but have completely different distribution assumptions. All simulations were conducted using R Statistical Software Version 4.3 [37]. This rigorous approach enabled us to comprehensively evaluate the effectiveness and robustness of our model and facilitate comparisons with other methods.

### 3.1. Data Generated from Hurdle Poisson Model

We began by applying our method to simulated data generated from the proposed hurdle Poisson model. Initially, all SNPs were assigned zero effect sizes. We then randomly selected a subset of *cis*-SNPs (ranging from 1 to 5) to have non-zero effect sizes, indicating true eQTL signals (eSNPs). These non-zero effect sizes were independently drawn from a normal distribution N(0,0.64). For each true eSNP, we used a multinomial distribution Mult(1;0.35,0.35,0.3) to determine whether its effect contributed solely to the presence of gene expression ($\beta^{L}\neq 0,\beta^{P}=0$), solely to the non-zero gene expression levels ($\beta^{L}=0,\beta^{P}\neq 0$), or to both ($\beta^{L}\neq 0,\beta^{P}\neq 0$). The intercept terms were set to 1, representing the baseline effect of gene expression aside from *cis*-SNP effects. Additionally, we included one covariate from real data, gender, whose effect size was also generated from N(0,0.64). We assumed its effect contributed to both the presence of gene expression and the non-zero gene expression levels, so $\omega^{L}=\omega^{P}$.

Following our proposed methodology, each indicator in the vector Z was generated from a Bernoulli distribution, $\mathrm{Bernoulli}(p_{gi})$, for gene *g* and cell *i*. The probability p of genes being expressed was determined using Equation (3), with effect sizes $\omega^{L}$ and $\beta^{L}$, and model matrices M and X, which included the intercept, gender, and all *cis*-SNPs. Each count value in the vector Y was then generated such that Y=0 if Z=0, and Y>0 if Z=1. The non-zero count values of Y were simulated from a zero-truncated Poisson distribution, $\mathrm{ZTP}(\mu_{gi})$, for gene *g* and cell *i*. The vector μ represents the mean non-zero expression levels calculated from Equation (4), using effect sizes $\omega^{P}$ and $\beta^{P}$, and the model matrices M and X, including the intercept, gender, and all *cis*-SNPs. To ensure robustness, we generated 100 different simulated datasets for each gene.

For each dataset, we conducted inference of BPHurdle using bootstrap hypothesis testing, as discussed in Section 2.3. In particular, we used the adjusted *p*-values from bootstrap hypothesis testing to determine the significance. Subsequently, we constructed a confusion matrix for each simulated dataset by comparing the results with the true values. A true positive (TP) was recorded when the estimate shared the same sign as the true value, and the null hypothesis $H_{0}:\beta_{gj}^{L}=\beta_{gj}^{P}=0$ was rejected. This stricter definition accounts for both statistical significance and correct directionality of genetic effects, which is important in eQTL interpretation where the sign of the effect (up- or down-regulation) is biologically meaningful. We note that this differs from the standard definition of TP based solely on rejection of the null hypothesis.

A true negative (TN) was tallied when the $H_{0}$ was not rejected, and the true value was zero. Conversely, a false negative (FN) was counted if the $H_{0}$ was not rejected, but the true value was not zero. Any estimate capable of rejecting the $H_{0}$ for a true value of zero or an estimate with a sign differing from the true value was categorized as a false positive (FP). Utilizing the confusion matrix from the analysis of each generated dataset, we computed sensitivity, specificity, and observed FDR as follows:

$$\mathrm{Sensitivity}=\frac{\mathrm{TP}}{\mathrm{TP}+\mathrm{FN}},$$

$$\mathrm{Specificity}=\frac{\mathrm{TN}}{\mathrm{TN}+\mathrm{FP}},$$

$$\mathrm{Observed}\;\mathrm{FDR}=\frac{\mathrm{FP}}{\mathrm{TP}+\mathrm{FP}}.$$

For each scenario, we determined the mean of these measurements across 100 simulated datasets.

To validate performance, we compared two versions of our model, BPHurdle with η=1 (denoted as BPHurdle_1_) and BPHurdle with η=0.5 (denoted as BPHurdle_0.5_), with other model frameworks, including the bootstrap penalized pure Poisson model with η=1 (denoted as BPPoisson_1_), the univariate hurdle Poisson regression model for each gene–SNP pair (denoted as SHurdle), the univariate pure Poisson regression model for each gene–SNP pair (denoted as SPoisson), CellRegMap [18], and MatrixEQTL [8] with average gene expression values across cells for each individual (i.e., pseudo-bulk model). While CellRegMap is primarily designed to model genetic effects along continuous cell states rather than in discrete cell-type settings, we include it here as a representative method that accounts for cell-state-dependent genetic effects; its performance in this setting should therefore be interpreted with caution. These methods were selected to represent a range of commonly used approaches in eQTL analysis, including univariate gene–SNP pair models, multivariable penalized models, pseudo-bulk approaches, and methods designed to capture different aspects of cellular heterogeneity, such as continuous cell-state frameworks.

For all methods, the significance level was defined as 0.05. Specifically, we performed FDR correction for the *p*-values across 100 simulated datasets using the Benjamini–Hochberg procedure [35], and if the adjusted *p*-value was less than 0.05, the SNP was considered significant.

**Figure 2:**
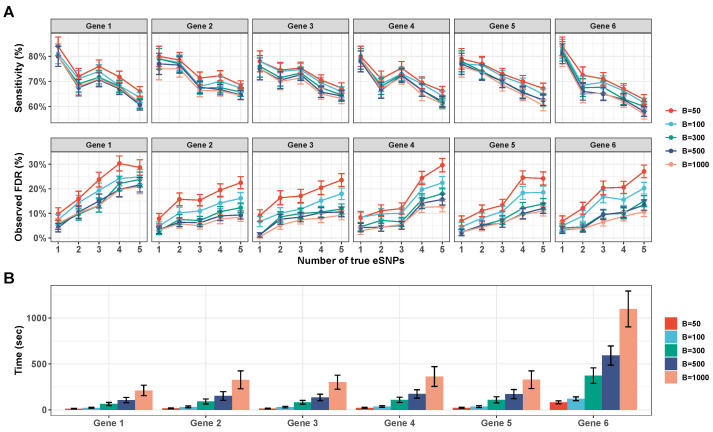
Results of BPHurdle with varying numbers of bootstrap samples (B=50,100,300,500,1000). (**A**) Sensitivity and false discovery rate (FDR) of BPHurdle_1_ as functions of the number of true eSNPs; different colored lines correspond to different values of bootstrap sample sizes. (**B**) Computational time of BPHurdle_1_ with different bootstrap sample sizes. Error bars represent standard errors across 100 simulated datasets.

Due to the specific bootstrap inference strategy used in our proposed model, the number of bootstrap samples, denoted as *B*, is an important factor that may affect both the accuracy of the inference and the computation speed. Therefore, we first investigated the performance of BPHurdle with a lasso penalty (i.e., η=1) across various values of *B* (50, 100, 300, 500, and 1000). It is well known that as the number of bootstrap samples increases, the variance should decrease. In Figure 2A, we observe that the sensitivity and FDR of BPHurdle both decrease as the number of bootstrap samples increases, although these changes have different implications: the decrease in FDR indicates improved control of false discoveries, whereas the decrease in sensitivity reflects a modest reduction in detection power. The rate of these changes gradually diminishes as *B* becomes larger. Additionally, performance differences across various numbers of SNPs considered in the model are minimal. For example, the model for Gene 1 includes 124 SNPs, while the model for Gene 6 includes 2857 SNPs (Table 1), yet their performances are similar. On the other hand, computational time increases exponentially with both the number of bootstrap samples and the number of SNPs included in the model (Figure 2B). Therefore, we conclude that B=100 is a good choice for fitting the model, as it balances good performance with reasonable computational time compared to larger values of *B*. In the remainder of this paper, we used B=100 for all bootstrapping strategies.

**Figure 3:**
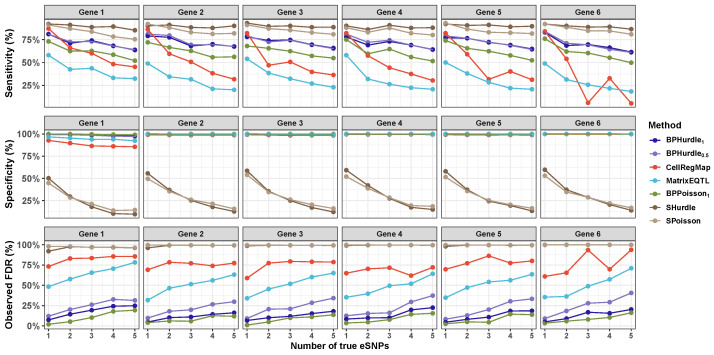
Performance of different eQTL mapping methods based on simulation data generated from the hurdle Poisson model. Panels show sensitivity, specificity, and observed false discovery rate (FDR) as functions of the number of true eSNPs, with different colors representing different methods.

Figure 3 illustrates the average sensitivity, specificity, and observed FDR for each method based on data generated from the proposed hurdle Poisson model. It is evident that as the number of SNPs affecting the specific gene increases, the performance of all approaches slightly decreases. However, our BPHurdle consistently demonstrates favorable performance compared to competing methods across all scenarios, with a sensitivity around 70%, specificity near 100%, and maintaining an observed FDR under or around 20%, as shown in Figure 3. Specifically, BPHurdle_1_ outperforms BPHurdle_0.5_, as expected, because BPHurdle_0.5_ imposes a looser elastic net penalty (η) compared to BPHurdle_1_, leading to more false discoveries. BPPoisson_1_ also exhibits good specificity and FDR control, though its sensitivity is lower than that of BPHurdle. In contrast, SHurdle, SPoisson, CellRegMap, and MatrixEQTL follow a univariate modeling approach to analyze one gene–SNP pair at a time, which remains common in current eQTL studies but results in higher FDRs. In particular, SHurdle and SPoisson exhibit the highest sensitivities but suffer from extremely high FDRs and low specificities, indicating a tendency to over-identify SNPs as significant, even when the number of true eSNPs is just one, which should align with their underlying assumption. CellRegMap performs well in sensitivity when a single true eSNP is present, but its performance declines as the number of eSNPs increases, and relatively high FDR is observed in this setting; we note that CellRegMap is primarily designed to model genetic effects along continuous cell states rather than in discrete cell-type settings, and its performance here should therefore be interpreted with caution. MatrixEQTL achieves high specificity but shows the lowest sensitivity and a relatively high FDR, indicating that while it effectively excludes non-significant SNPs, it frequently fails to identify true regulatory variants. Overall, these results demonstrate that BPHurdle achieves a favorable balance between sensitivity and false discovery control across a range of scenarios.

**Figure 4:**
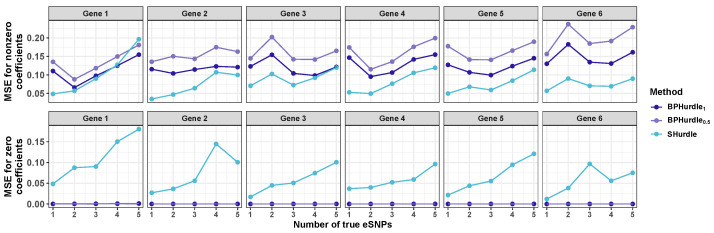
MSE of different eQTL mapping methods based on simulation data generated from the hurdle Poisson model. Panels show MSE for non-zero and zero coefficients, respectively, as functions of the number of true eSNPs, with different colors representing different methods. The Y-axis is shown on a linear scale.

Because BPHurdle_1_, BPHurdle_0.5_, and SHurdle are methods that utilize the same two-part model as the data assumption in this simulation, we can assess the precision of inference by examining the mean square errors (MSEs) for non-zero coefficients and zero coefficients separately (Figure 4). MSE is computed on the original scale of the regression coefficients, where coefficients from both the logistic and zero-truncated Poisson components are pooled together and grouped according to whether their true values are zero or non-zero. As observed, for BPHurdle_1_ and BPHurdle_0.5_, a stronger elastic net penalty (η) leads to lower MSEs. While SHurdle exhibits the lowest MSE for non-zero coefficients, its MSE for zero coefficients is significantly higher compared to BPHurdle. This observation aligns with our findings in Figure 3, indicating that SHurdle tends to identify too many SNPs as significant, thus resulting in high MSEs for zero coefficients. These results suggest that BPHurdle provides more balanced estimation accuracy, particularly in controlling errors for null effects, which is consistent with its strong FDR performance.

To further assess the representativeness of the simulation design, we conducted additional simulations by including genes from multiple chromosomes (chromosomes 1, 7, and 12). The results show broadly consistent performance patterns across different genomic contexts, suggesting that the proposed method is reasonably robust to variations in chromosome-specific SNP density and LD structure (Supplementary Figure S1).

We also evaluated the sensitivity of the proposed method to the number of cells per individual (e.g., 50, 100, and 200 cells). As the number of cells increases, sensitivity improves, while FDR also increases, reflecting a trade-off between detection power and false discovery control, where more signals, including weaker or borderline effects, are detected. This highlights the impact of sample size on inference performance and is consistent with increased statistical power in high-dimensional settings. Detailed results are provided in Supplementary Figure S2.

### 3.2. Data Generated from Zero-Inflated Negative Binomial Model

In the second simulation study, we utilized a different distribution to simulate data and evaluate the robustness of our proposed method. We generated data using the zero-inflated negative binomial model, whose probability mass function is as follows:

$$\mathrm{Pr}\left(Y_{gi}=y\right)=\left\{\begin{array}{cc} \left(1-p_{gi}\right)+p_{gi}{\left(\frac{\phi_{g}}{\mu_{gi}+\phi_{g}}\right)}^{\phi_{g}}, & y=0,\\ p_{gi}\frac{\Gamma (y+\phi_{g})}{y!\Gamma (\phi_{g})}{\left(\frac{\mu_{gi}}{\mu_{gi}+\phi_{g}}\right)}^{y}{\left(\frac{\phi_{g}}{\mu_{gi}+\phi_{g}}\right)}^{\phi_{g}}, & y>0, \end{array}\right.$$

where $p_{gi}$ and $\mu_{gi}$ depended on the same sets of coefficients, ω and β, and model matrices, M and X, were generated in Section 3.1 through Equations (3) and (4). Additionally, the dispersion parameter $\phi_{g}>0$ was generated from a $\mathrm{Lognormal}(\gamma_{1},\gamma_{2})$ distribution, with the estimates of $\gamma_{1}$ and $\gamma_{2}$ derived from the real scRNA-seq data.

**Figure 5:**
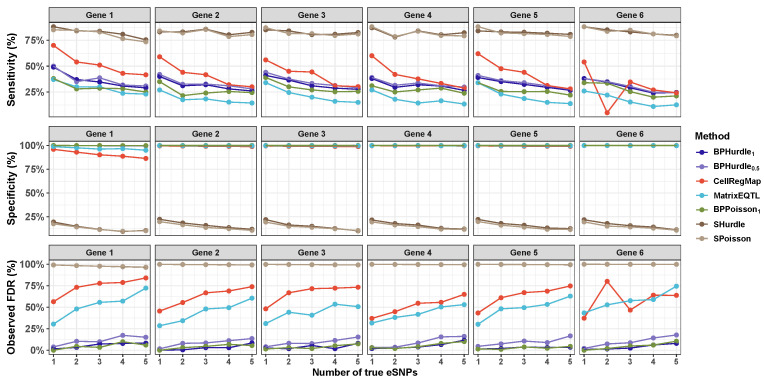
Results of different eQTL mapping methods based on simulation data generated from the zero-inflated negative binomial model. Panels show sensitivity, specificity, and observed false discovery rate (FDR) as functions of the number of true eSNPs, with different colors representing different methods. Compared with the hurdle Poisson scenario, all methods exhibit lower sensitivity under the zero-inflated negative binomial setting, likely due to the additional overdispersion and variability in gene expression.

Figure 5 shows that although the sensitivity of all methods decreases compared to Figure 3, the overall performance trends remain consistent. Notably, BPHurdle_0.5_ still exhibits a higher FDR compared to BPHurdle_1_, especially as the number of true eSNPs increases. The three multivariable regression models, BPHurdle_1_, BPHurdle_0.5_, and BPPoisson_1_, demonstrate lower FDR and sensitivity compared to Figure 3, indicating that they become more conservative under this zero-inflated setting and are more likely to infer no regulatory effect. Although CellRegMap achieves better sensitivity than the multivariable models in this context, it continues to suffer from a very high FDR. Meanwhile, MatrixEQTL consistently performs poorly relative to other methods, with the lowest sensitivity and high FDR. It is worth noting that while the two gene–SNP pair models, SHurdle and SPoisson, maintain a good sensitivity of around 80%, their specificities are only around 20%, with observed FDRs close to 100% even when there is only one true eSNP. In other words, these models make many more type I errors (1—Specificity) and false discoveries compared to others. Taken together, these results indicate that BPHurdle remains stable under more complex data settings, maintaining a reasonable balance between sensitivity and false discovery control despite increased model misspecification.

## 4. Case Study

We applied our methods to the scRNA-seq data of 259 individuals from a Peruvian tuberculosis (TB) progression cohort [38] and integrated it with genotype data from the same individuals from a previous study [39]. The genotype data includes 1,353,840 autosomal variants after preprocessing (Appendix A). The scRNA-seq data contains 500,089 cells and 6526 genes, after filtering out genes not expressed in at least 95% of the cells. We first performed differential expression analysis on the scRNA-seq data to compare TB cases and controls using MAST [27]. We identified 11 significantly differentially expressed genes (with an FDR-adjusted *p*-value less than 0.05 and an estimated absolute $\log_{2}$ fold change greater than 0.05) for subsequent eQTL mapping. As an example, we focused on three specific CD4+ helper T cell subsets: CD4+ Th1 (37,566 cells), CD4+ Th2 (20,696 cells), and CD4+ Th17 (16,987 cells), separately.

Considering the characteristics of single-cell data, we incorporate the cellular detection rate (CDR) into the model, treating it as a covariate within M in Equations (3) and (4). The CDR for cell i(i=1,2,…,n) is defined as:

$$CDR_{i}=\frac{1}{G}\sum_{g=1}^{G}Z_{gi}.$$

**Figure 6:**
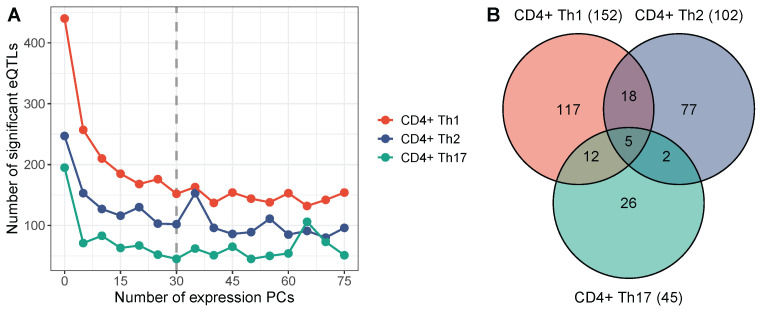
Number of significant eQTLs for the 11 differentially expressed genes. (**A**) Number of significant eQTLs across different numbers of gene expression principal components (PCs) used as covariates for modeling. The dashed vertical line marks 30 PCs, beyond which the number of significant eQTLs remained relatively stable. (**B**) Number of significant eQTLs unique to or shared between CD4+ cell subtypes Th1, Th2, and Th17, with 30 gene expression PCs in the model.

This variable represents the proportion of genes expressed in each cell and can effectively account for both technical and biological factors that globally influence gene expression [27]. In addition to CDR, the model was adjusted for age, sex, batch effect, the first five principal components (PCs) of genotype dosage values, and the top 30 PCs of gene expression values. Genotype PCs were used to account for residual population structure among individuals of Peruvian genetic ancestry. Gene expression PCs were calculated within each cell type to regress out the nongenetic structure in the data. Figure 6A shows that the top 30 expression PCs stabilize the eQTL discovery process.

The numbers of eSNPs for the CD4+ cell subtypes Th1, Th2, and Th17 are shown in Figure 6B. As we can see, the number of eSNPs varies significantly among these three cell types, indicating the presence of cell-type-specific eQTLs, which matches our expectations. The eQTL results are very different for different differentially expressed genes (Table 2).

In this study, we analyzed the eGene *IL32* as an example. *IL32* encodes Interleukin 32, a member of the cytokine family, whose expression increases following the activation of T cells [40]. Several studies have demonstrated that *IL32* serves as a molecular biomarker with protective effects against TB [41,42,43]. However, *IL32* expression is down-regulated in TB patients compared to controls, suggesting that *IL32* may fail to activate properly in individuals with TB, thereby impairing the immune response against the disease.

Our eQTL analysis, conducted using BPHurdle with η=1, identified 41 eSNPs that significantly regulate *IL32*. These variants should be interpreted as candidate regulatory loci identified under a multivariable framework, rather than independently validated causal variants. Among these, 22 eQTLs are specific to CD4+ Th1 cells, 21 to CD4+ Th2 cells, and 9 to CD4+ Th17 cells. Notably, the variant rs8052002 exerts a significant down-regulatory effect on *IL32* non-zero expression (with estimated effects of $\hat{\beta }=-0.094$ for CD4+ Th1 cells and $\hat{\beta }=-0.142$ for CD4+ Th2 cells), whereas rs28372698 has a significant up-regulatory effect on *IL32* non-zero expression ($\hat{\beta }=0.059$ for CD4+ Th17 cells). These results align with findings from the GTEx database [44], where the normalized effect size of rs8052002 on *IL32* is −0.097 in whole blood, and the average normalized effect size of rs28372698 on *IL32* is 0.192 across 12 different tissues. These results highlight the cell-type-specific regulatory mechanisms of *IL32*, which may play distinct roles in modulating immune responses across T cell subtypes. While only a subset of identified variants could be directly validated in external databases, several *IL32* eSNPs identified by BPHurdle were also reported in GTEx, eQTLGen, and DICE (Supplementary Tables S3–S5). In particular, rs8052002 showed consistent associations in both GTEx and eQTLGen, while several additional variants identified in CD4+ Th2 and Th17 cells were supported by immune-related eQTL resources. Nevertheless, many variants were not directly observed in external databases, which is expected given differences in tissue composition, disease context, population structure, statistical modeling strategies, and cell-type resolution between bulk and single-cell datasets. These findings suggest that BPHurdle is capable of identifying biologically relevant regulatory variants while also capturing potential cell-type-specific signals that may not be detectable in bulk-level analyses.

Biologically, these findings suggest that genetic variation may contribute to the dysregulation of *IL32* expression observed in TB patients, potentially affecting downstream immune responses. Given the established role of *IL32* in modulating immune activity and host defense, the identified eQTLs may influence disease progression by altering the regulation of *IL32* across different CD4+ T cell subtypes.

Interestingly, some variants exhibit different directions of effect in CD4+ Th17 cells compared with Th1 and Th2 cells. This pattern may reflect subtype-specific regulatory mechanisms, as Th17 cells are known to play distinct roles in immune responses to tuberculosis [45,46]. In particular, IL-17–producing CD4+ T cells are enriched at sites of infection and contribute to host immune control, while their dysregulation has been associated with disease progression. In addition, *IL32* has been implicated in modulating the balance between Th1 and Th17 immune responses [43], suggesting that the observed cell-type-specific regulatory effects may reflect context-dependent genetic control of immune pathways. These findings should therefore be interpreted as hypothesis-generating and warrant further experimental validation.

To further assess the information provided by BPHurdle, we compared these results with a pseudo-bulk eQTL analysis on the same TB data using MatrixEQTL. Across the 11 genes analyzed, BPHurdle identified eSNPs for most genes (Table 2), whereas MatrixEQTL detected associations for only three genes (Supplementary Table S2). This difference is consistent with the ability of BPHurdle to accommodate zero-inflated and sparse expression patterns, which are not well captured by standard linear models. In addition, MatrixEQTL requires the use of pseudo-bulk expression summaries, which aggregate expression measurements across cells or spatial locations and may obscure cell-level heterogeneity. Notably, for *IL32*, MatrixEQTL identified a substantially larger number of eSNPs compared to BPHurdle. This may be attributed to the relatively high and less sparse expression of *IL32*, which better satisfies the assumptions of linear models and increases statistical power. In addition, the large number of detected eSNPs may reflect aggregated marginal associations across cells. In contrast, BPHurdle identified a more targeted set of variants and revealed distinct cell-type-specific regulatory patterns, highlighting its ability to capture heterogeneity that may be masked in pseudo-bulk analyses. Detailed results and additional analyses are provided in Supplementary Section S1.3.

## 5. Discussion

In this paper, we proposed a bootstrap penalized hurdle regression model with a Poisson distribution to map cell-type-specific *cis*-eQTLs using scRNA-seq data. The hurdle model effectively captures the unique properties of single-cell data, such as the abundance of zero counts and skewed expression patterns, and it can identify the concurrent effects of multiple genetic variants on a single gene. This strategy can reduce false discoveries compared to current approaches that analyze single gene–SNP pairs, as demonstrated in our simulation studies (Section 3). Despite these advantages, several limitations and potential avenues for improvement of the proposed framework warrant discussion.

A key limitation of the proposed framework is the assumption that cells from the same donor are independent. In practice, cells from a single individual share both genetic background and environmental influences, leading to potential intra-donor correlation. Ignoring this correlation may affect variance estimation and, consequently, statistical inference. While our current model does not explicitly account for this dependency structure, incorporating donor-level random effects or other strategies to model within-donor correlation represents an important direction for future methodological development.

We note that the proposed framework adopts a Poisson distribution within the hurdle model, which may be restrictive given the overdispersed nature of scRNA-seq data. Diagnostic analysis using randomized quantile residuals [47] suggests deviations from the Poisson assumption, indicating that variability in gene expression is not fully captured (Supplementary Figure S3). Nevertheless, in our setting the Poisson specification serves as a working model within a penalized multivariate framework, where the primary objective is variable selection rather than full probabilistic modeling. Empirically, the proposed method remains robust under more complex data-generating mechanisms, as shown in simulation studies under ZINB settings, suggesting reasonable stability under model misspecification. Extensions to more flexible distributions such as the Negative Binomial represent a natural direction for future work.

We introduced penalization into the model to identify true genetic effect signals from noisy data, typically involving hundreds of SNPs. When considering several SNPs within a specific window size, linkage disequilibrium (LD) becomes an unavoidable issue. Although LD pruning can be performed during preprocessing, remaining SNPs can still be correlated if they are outside the window size used for pruning or have correlation coefficients close to the threshold. Additionally, the genotype dosage matrix, which contains the values 0, 1, and 2 representing the number of alternative alleles of the SNP, can lead to severe linear combination issues and singularity in the data matrix. These factors make traditional multiple generalized linear regression approaches difficult to apply, making penalization a natural choice to address these challenges. However, simple penalization may still cause issues, such as randomly selecting one SNP as the eSNP and ignoring others, which may mask latent effects within a group of correlated SNPs. Future research should focus on more comprehensive considerations of group penalties (e.g., group lasso [48], sparse group lasso [49]), which can better account for correlated SNP groups, or SNP co-expression networks.

To obtain inferences from the penalized model, we used bootstrap hypothesis testing. Although our results in Figure 2 show that a bootstrap sample size of B=100 provides good performance without excessive computational time, the computational speed remains a concern if the number of cells in the model is extremely high. The average computational time (standard deviation) in the TB case study was 65.40 (28.85) minutes, 37.60 (15.58) minutes, and 31.11 (13.07) minutes across 11 differentially expressed genes for CD4+ Th1, Th2, and Th17, respectively, on HiPerGator using a single core of an AMD EPYC 75F3 32-Core Processor and 60 GB of RAM. These results suggest that the computational cost increases with the number of cells, SNPs, and bootstrap samples, and may become substantial for large-scale genome-wide analyses. Therefore, it is important to explore other inference approaches that can offer good estimates for the penalized model and operate faster than bootstrapping, ensuring the scalability of solving eQTL problems. Possible strategies to improve computational efficiency include parallelization across genes or bootstrap samples, accelerated bootstrap methods such as the Bag of Little Bootstraps (BLB) [50], and approximate inference techniques.

We also note that hyperparameters in the elastic-net penalty are selected via cross-validation prior to bootstrap inference, which introduces a degree of data reuse. To further evaluate the potential impact of this issue, we conducted an additional sensitivity analysis comparing cross-validated and fixed hyperparameters in one simulation setting. The fixed hyperparameters were estimated independently using a separate subset of simulated datasets, while performance evaluation was conducted on the remaining datasets. Compared with the fixed-hyperparameter approach, the cross-validated approach achieved similar sensitivity but slightly lower specificity and higher observed FDR (Supplementary Figure S4). These findings suggest that data reuse during hyperparameter selection may introduce a modest optimistic bias in inference, although the overall performance patterns remained similar in the evaluated setting. More rigorous strategies, such as nested cross-validation or sample splitting, could further address this issue but would substantially increase computational cost.

In downstream colocalization analysis, researchers often use *p*-values to investigate associations between GWAS and eQTLs. Although we can obtain *p*-values from bootstrap hypothesis testing, the nature of penalized models results in *p*-values that are almost binary after FDR correction, meaning the values are very close to 0 or 1. The lack of gradation makes it difficult to perform correlation analysis between the *p*-values of GWAS and eQTLs. This phenomenon is related to broader challenges in statistical inference following variable selection, which have been extensively studied in the literature [51,52]. However, extending these approaches to penalized hurdle models with bootstrap-based inference is nontrivial and beyond the scope of the current work. Therefore, developing more appropriate inference procedures for penalized generalized linear models under complex data distributions represents another valuable research direction.

In our proposed model, we considered the main effects of SNPs on genes. For cell-type-specific eQTL analysis, the model needs to be fitted separately for each cell type. Although this approach straightforwardly identifies cell-type-specific eQTL signals, some researchers have found that regulatory effects may vary along continuous cell states and have proposed more complex model structures to account for this phenomenon, such as using interaction terms, as implemented in recent methods for modeling continuous cellular states (e.g., CellRegMap and related approaches) [17,18]. Given the complexity already present in our model, we have not incorporated these factors, but it will be imperative to adapt our method to various scenarios and extend its applicability in future studies.

In general, our innovative method bridges gaps in current eQTL mapping methods and provides a comprehensive model for uncovering concurrent eQTL signals for specific genes. It offers a potential template to solve the corresponding eQTL problems. Future studies can investigate the applicability of our model to more complex structures and further confirm its effectiveness in more real-world scenarios.

## Figures and Tables

**Table 1:** Gene information used in simulation studies.

	Gene ID	Chromosome	Number of *cis*-SNP
Gene 1	ENSG00000198885	2	124
Gene 2	ENSG00000119844	2	607
Gene 3	ENSG00000072182	2	558
Gene 4	ENSG00000279490	16	834
Gene 5	ENSG00000205084	16	784
Gene 6	ENSG00000103187	16	2857

**Table 2:** Number of eSNPs for CD4+ cell subtypes Th1, Th2, and Th17 for the 11 differentially expressed genes, with 30 gene expression principal components in the model.

Gene	Chromosome	Width	Number of *cis*-SNPs	Number of Th1 eSNPs	Number of Th2 eSNPs	Number of Th17 eSNPs
*CD69*	12	8416	2366	5	5	4
*DUSP1*	5	3100	2459	8	2	0
*FOS*	14	3405	2629	21	10	4
*GZMH*	14	3220	2912	0	2	7
*IL32*	16	16,896	2517	22	21	9
*JUN*	1	3257	2286	9	5	5
*JUNB*	19	1830	2634	37	33	11
*JUND*	19	1929	2723	24	8	4
*KLF6*	10	9286	3627	15	10	1
*NKG7*	19	1096	3400	0	0	0
*SOCS3*	17	3300	3603	11	6	0

## Data Availability

The data used in this study are available from the Database of Genotypes and Phenotypes (dbGaP) under accession numbers phs002025 and phs002467, subject to dbGaP data access approval. The code scripts utilized in this study are available at https://github.com/dongyuanwu/BPHurdle, accessed on 7 May 2026.

## Supplementary Materials

The following supporting information can be downloaded at: https://www.mdpi.com/article/10.3390/genes17060625/s1, Figure S1: Performance of BPHurdle based on simulation data generated from genes sampled across multiple chromosomes (chromosomes 1, 7, and 12). Panels show sensitivity, specificity, and observed false discovery rate (FDR), with different colors representing different methods; Figure S2: Performance of BPHurdle under varying numbers of cells per individual. Panels show sensitivity, specificity, and observed false discovery rate (FDR) as functions of the number of cells, with different colors representing different cell count settings; Figure S3: QQ plot of randomized quantile residuals for the non-zero component of the Poisson hurdle model applied to the TB dataset. The reference line corresponds to the standard normal distribution; Figure S4: Performance of BPHurdle under sensitivity analysis for hyperparameter selection in the primary simulation settings. Panels show sensitivity, specificity, and observed false discovery rate (FDR) comparing cross-validated and fixed hyperparameters, with different colors representing different approaches. Fixed hyperparameters were estimated using the first 20 simulated datasets, while performance evaluation was conducted on the remaining 80 datasets; Table S1: Gene information used in extended simulation studies; Table S2: Number of eSNPs for CD4+ cell subtypes Th1, Th2, and Th17 for the 11 differentially expressed genes using MatrixEQTL; Table S3: External validation of *IL32* eSNPs identified by BPHurdle in CD4+ Th1 cells using publicly available eQTL resources, including GTEx, eQTLGen, and DICE; Table S4: External validation of *IL32* eSNPs identified by BPHurdle in CD4+ Th2 cells using publicly available eQTL resources, including GTEx, eQTLGen, and DICE; Table S5: External validation of *IL32* eSNPs identified by BPHurdle in CD4+ Th17 cells using publicly available eQTL resources, including GTEx, eQTLGen, and DICE.

## Appendix A. Preprocessing Steps for Genotype Data in Case Study

The original genotype data, in PLINK bed/bim/fam file format, contains 712,534 variants from 4002 Peruvian donors enrolled in a previous study. The 259 donors with single-cell data used in our case study (Section 4) are a subset of these 4002 donors. We performed all preprocessing steps, including quality control, leftover, pre-phasing, and imputation, on the original 4002 Peruvian donors and then extracted the subset of information that could be matched to the single-cell data.

First, we used PLINK 2.0 (PLINK2) [53] for quality control, focusing only on autosomes (chromosomes 1 to 22). Based on the original genotype data, we filtered out variants with low call rates (<95%) and Hardy–Weinberg *p*-values less than $1\times 10^{-6}$. Additionally, we removed duplicated variants, retaining only their first instance. After quality control, 679,262 genotyped variants remained.

For imputation, we used SHAPEIT4 v4.2.2 [54] to pre-phase genotypes and IMPUTE5 v1.1.5 [55] to impute genotypes, using the 1000 Genomes Project Phase 3 [36] as the reference panel. We then conducted another round of quality control on the imputed genotype data to remove individuals without scRNA-seq data and filter out variants with an IMPUTE-INFO score less than 0.9, minor allele frequency (MAF) less than 0.01, or deletions.

To maintain consistent genome assembly with the scRNA-seq data, we converted the remaining variants from b37/GRCh37 to Hg38/GRCh38 using Picard LiftoverVcf. For principal component analysis (PCA), we used PLINK2 for LD pruning to remove highly correlated variants ($r^{2}>0.2$, –indep-pairwise 50 5 0.2) from the lifted genotype data, and then conducted PCA using PLINK2 (–pca).

For the downstream analysis, we did not perform LD pruning to avoid losing too much information. In the final round of quality control, we filtered out variants with MAF of less than 0.05 and those with one or more multi-character allele codes. Finally, the processed genotype data, consisting of 259 donors and 1,353,840 autosomal variants, was used for the eQTL analysis.

## Appendix B. Derivation of Equation (5)

Based on Equations (1)–(4), the corresponding log-likelihood function is

$$\begin{array}{cc} \; & l(\beta_{g}^{L},\beta_{g}^{P},\omega_{g}^{L},\omega_{g}^{P})\\ = & \sum_{i:y_{gi}=0}\log\left\{\frac{1}{1+\exp(m_{i}\omega_{g}^{L}+x_{gi}\beta_{g}^{L})}\right\}+\sum_{i:y_{gi}>0}\log\left\{\frac{\exp(m_{i}\omega_{g}^{L}+x_{gi}\beta_{g}^{L})}{1+\exp(m_{i}\omega_{g}^{L}+x_{gi}\beta_{g}^{L})}\right\}\\ \; & +\sum_{i:y_{gi}>0}\left[y_{gi}\left(m_{i}\omega_{g}^{P}+x_{gi}\beta_{g}^{P}\right)-\exp\left(m_{i}\omega_{g}^{P}+x_{gi}\beta_{g}^{P}\right)-\log\left(y_{gi}!\right)\right]\\ \; & -\sum_{i:y_{gi}>0}\log\left\{1-\exp(-\exp\left(m_{i}\omega_{g}^{P}+x_{gi}\beta_{g}^{P}\right))\right\}\\ = & \sum_{i=1}^{n}\left[z_{gi}\left(m_{i}\omega_{g}^{L}+x_{gi}\beta_{g}^{L}\right)-\log\left\{1+\exp(m_{i}\omega_{g}^{L}+x_{gi}\beta_{g}^{L})\right\}\right]\\ \; & +\sum_{i:y_{gi}>0}\left[y_{gi}\left(m_{i}\omega_{g}^{P}+x_{gi}\beta_{g}^{P}\right)-\exp\left(m_{i}\omega_{g}^{P}+x_{gi}\beta_{g}^{P}\right)-\log\left(y_{gi}!\right)\right.\\ \; & \;\;\;\;\;\;\;\;\;\;\;\;\;\;-\left.\log\left\{1-\exp(-\exp\left(m_{i}\omega_{g}^{P}+x_{gi}\beta_{g}^{P}\right))\right\}\right]\\ = & l_{1}\left(\beta_{g}^{L},\omega_{g}^{L}\right)+l_{2}\left(\beta_{g}^{P},\omega_{g}^{P}\right), \end{array}$$

where $m_{i}$ and $x_{gi}$ are the *i*-th row vectors of M and $X_{g}$, respectively. In addition, $l_{1}\left(\beta_{g}^{L},\omega_{g}^{L}\right)$ and $l_{2}\left(\beta_{g}^{P},\omega_{g}^{P}\right)$ are the log-likelihood functions of the logistic regression model and the zero-truncated Poisson regression model, separately.
