# Supplementary material for: Identifying Single-Cell Expression Quantitative Trait Loci Using a Bootstrap Penalized Hurdle Model
Source: Genes (Basel). 2026 May 29;17(6):625. doi: 10.3390/genes17060625 (PMC13299111; doi:10.3390/genes17060625)
Supplement: Supplementary file 1 [file genes-17-00625-s001.zip › genes-4262845-supplementary.pdf]

# Supplementary Materials for Identifying Single-cell Expression Quantitative Trait Loci using a Bootstrap Penalized Hurdle Model

## Contents


**S1 Supplementary Notes** **2**

S1.1 Extended Simulation Across Multiple Chromosomes . . . . . . . . . . . . . . . 2

S1.2 Sensitivity Analysis With Respect to the Number of Cells per Individual . . 3

S1.3 Case Study Using MatrixEQTL . . . . . . . . . . . . . . . . . . . . . . . . . 4

S1.4 Sensitivity Analysis for Hyperparameter Selection . . . . . . . . . . . . . . . . 5

**S2 Supplementary Figures** **6**

**S3 Supplementary Tables** **8**

# S1 Supplementary Notes

## S1.1 Extended Simulation Across Multiple Chromosomes

To assess the robustness and generalizability of the proposed method with respect to genomic context, we extended the simulation design to include genes sampled from additional chromosomes beyond those considered in the main text. In addition to chromosomes 2 and 16, we randomly selected genes from chromosomes 1, 7, and 12, thereby incorporating a broader range of linkage disequilibrium (LD) structures and SNP densities. The gene information used in the extended simulations is detailed in Supplementary Table S1.

For each selected gene, cis-SNPs within the same genomic window were used to construct the genotype matrix, resulting in varying numbers of SNPs per gene, consistent with realistic genomic settings. The simulation framework, including data generation and parameter settings, remained the same as described in the main text to ensure comparability.

The performance of BPHurdle across these extended scenarios is summarized in Supplementary Figure S1, which presents sensitivity, specificity, and FDR for genes sampled from different chromosomes. As shown in the figure, BPHurdle maintains consistent performance across genes from diverse genomic regions, despite substantial variability in SNP density and LD structure.

Overall, these results suggest that the proposed method is robust to chromosome-specific genomic characteristics and generalizes well across different genomic contexts.

## S1.2 Sensitivity Analysis With Respect to the Number of Cells per Individual

In the main simulation study, we assumed that each individual possesses 100 cells as a simplifying setting. To evaluate the sensitivity of the proposed method to this parameter, we conducted additional simulations under varying numbers of cells per individual.

Specifically, we considered multiple settings with different cell counts (50, 100, and 200 cells per individual), while keeping other simulation parameters unchanged. For each setting, we evaluated model performance using sensitivity, specificity, and FDR.

The results are summarized in Supplementary Figure S2, which shows the relationship between model performance and the number of cells per individual. As illustrated, increasing the number of cells leads to improved sensitivity, reflecting enhanced statistical power to detect true associations. At the same time, FDR shows a moderate increase, likely due to the detection of weaker signals as the effective sample size grows. Specificity remains relatively stable across different settings.

Despite these changes, BPHurdle maintains a favorable balance between sensitivity and false discovery control across all scenarios, indicating that the method is robust to variations in the number of cells per individual.

These findings highlight the importance of cellular sample size in single-cell eQTL studies and provide practical guidance for applying the proposed method to datasets with varying cell counts.

## S1.3 Case Study Using MatrixEQTL

To benchmark BPHurdle against a widely used eQTL mapping approach, we applied MatrixEQTL to the same dataset and preprocessing pipeline. For both methods, we analyzed the same set of 11 genes, SNPs, and covariates, and restricted the analysis to cis-SNPs within a predefined genomic window. Statistical significance was determined using the same false discovery rate (FDR) threshold.

As MatrixEQTL requires continuous expression input, it was applied to pseudo-bulk expression data obtained by aggregating expression counts across cells for each sample. In contrast, BPHurdle was applied directly to the original count-level data, preserving both sparsity and cell-level variability.

The number of detected eSNPs for each gene using MatrixEQTL is summarized in Supplementary Table S2. BPHurdle identified at least one significant eSNP for 10 genes (Table 2 in the main text), whereas MatrixEQTL detected associations for only three genes. This difference suggests that BPHurdle is more sensitive to regulatory signals in genes with sparse and zero-inflated expression patterns, which may be attenuated when using pseudo-bulk aggregation and linear modeling.

Notably, for *IL32*, MatrixEQTL identified a substantially larger number of eSNPs compared to BPHurdle. One possible explanation is that *IL32* exhibits relatively high and less sparse expression, making it more robust to aggregation and more compatible with the assumptions of linear models. As a result, the statistical power of MatrixEQTL may be enhanced for this gene. In addition, pseudo-bulk aggregation may amplify differences in expression across samples, potentially leading to stronger association signals. The large number of detected eSNPs may also reflect clusters of correlated SNPs in linkage disequilibrium rather than independent regulatory effects.

In contrast, BPHurdle explicitly models both the presence/absence and magnitude of gene expression at the count level, providing a more nuanced characterization of regulatory effects in sparse data settings. These results highlight the importance of modeling choices and data representation in eQTL analysis, particularly for zero-inflated transcriptomics data.

## S1.4 Sensitivity Analysis for Hyperparameter Selection

To further evaluate the potential impact of data reuse during hyperparameter selection, we conducted an additional sensitivity analysis comparing cross-validated and fixed hyperparameters in the primary simulation settings. The fixed hyperparameters were estimated independently using a separate subset of simulated datasets (the first 20 datasets), while performance evaluation was conducted on the remaining 80 datasets. Supplementary Figure S4 summarizes the comparison results. Compared with the fixed-hyperparameter approach, the cross-validated approach achieved similar sensitivity but slightly lower specificity and higher observed FDR, suggesting a modest optimistic bias introduced by data reuse during hyperparameter selection.

# S2 Supplementary Figures

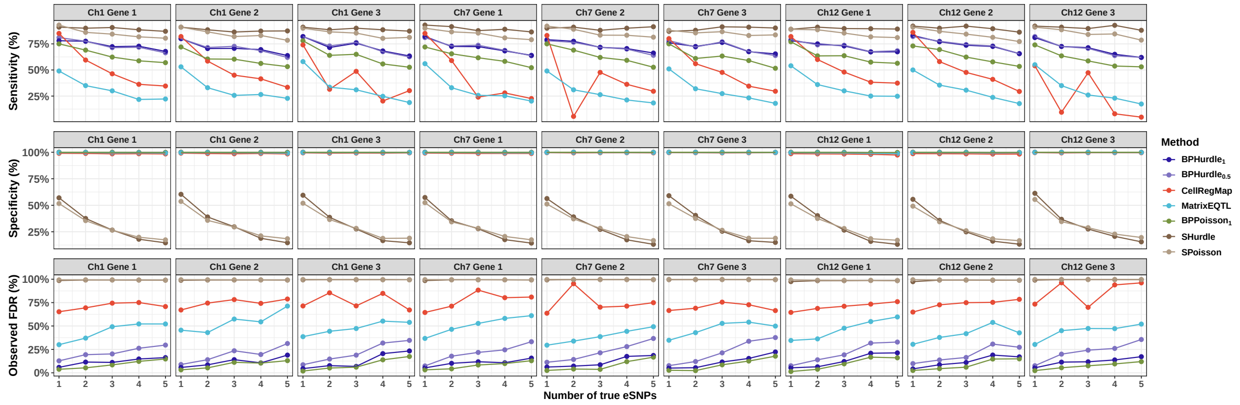


Supplementary Figure S1: Performance of BPHurdle based on simulation data generated from genes sampled across multiple chromosomes (chromosomes 1, 7, and 12). Panels show sensitivity, specificity, and observed false discovery rate (FDR), with different colors representing different methods.

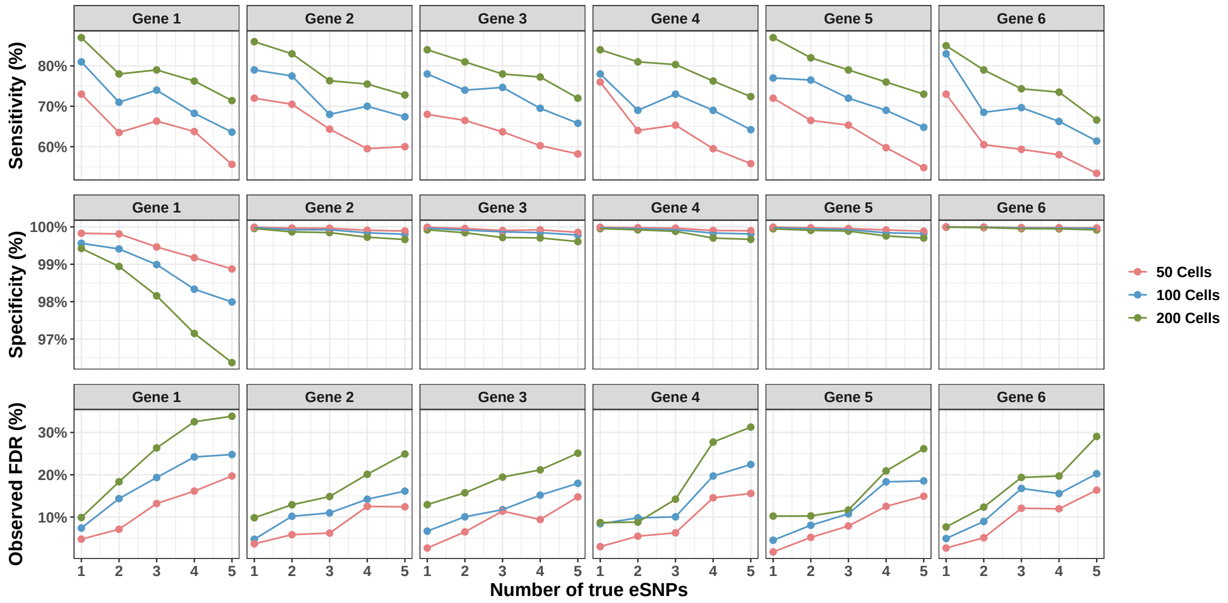


Supplementary Figure S2: Performance of BPHurdle under varying numbers of cells per individual. Panels show sensitivity, specificity, and observed false discovery rate (FDR) as functions of the number of cells, with different colors representing different cell count settings.

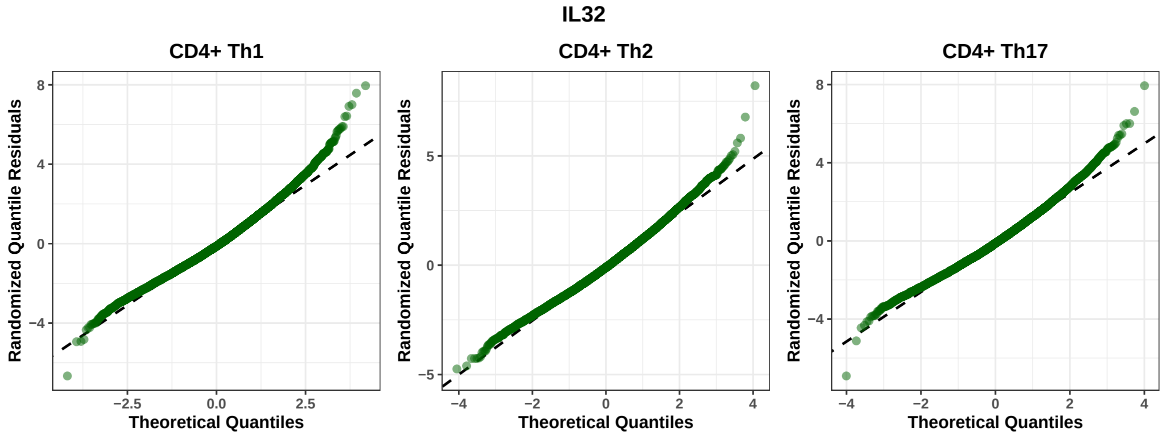


Supplementary Figure S3: QQ plot of randomized quantile residuals for the non-zero component of the Poisson hurdle model applied to the TB dataset. The reference line corresponds to the standard normal distribution.

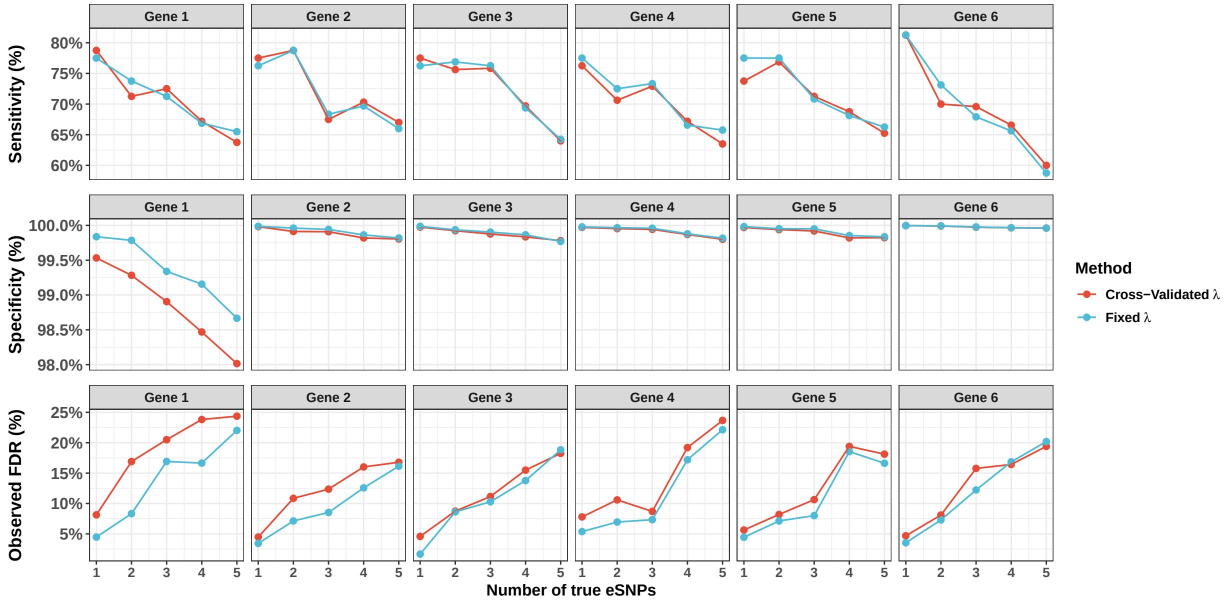


Supplementary Figure S4: Performance of BPHurdle under sensitivity analysis for hyperparameter selection in the primary simulation settings. Panels show sensitivity, specificity, and observed false discovery rate (FDR) comparing cross-validated and fixed hyperparameters, with different colors representing different approaches. Fixed hyperparameters were estimated using the first 20 simulated datasets, while performance evaluation was conducted on the remaining 80 datasets.

# S3 Supplementary Tables

Supplementary Table S1: Gene information used in extended simulation studies.

| Chromosome | Gene | Gene ID | Number of *cis*-SNP |
|---|---|---|---|
| 1 | Gene 1 | ENSG00000188396 | 504 |
| 1 | Gene 2 | ENSG00000156876 | 577 |
| 1 | Gene 3 | ENSG00000143125 | 855 |
| 7 | Gene 1 | ENSG00000222011 | 663 |
| 7 | Gene 2 | ENSG00000285480 | 1141 |
| 7 | Gene 3 | ENSG00000133627 | 1173 |
| 12 | Gene 1 | ENSG00000111602 | 346 |
| 12 | Gene 2 | ENSG00000111371 | 438 |
| 12 | Gene 3 | ENSG00000177192 | 1328 |

Supplementary Table S2: Number of eSNPs for CD4+ cell subtypes Th1, Th2, and Th17 for the 11 differentially expressed genes using MatrixEQTL.

| Gene | Chromosome | Width | Number of *cis*-SNPs | Number of Th1 eSNPs | Number of Th2 eSNPs | Number of Th17 eSNPs |
|---|---|---|---|---|---|---|
| *CD69* | 12 | 8416 | 2366 | 0 | 0 | 0 |
| *DUSP1* | 5 | 3100 | 2459 | 0 | 0 | 0 |
| *FOS* | 14 | 3405 | 2629 | 0 | 0 | 0 |
| *GZMH* | 14 | 3220 | 2912 | 0 | 0 | 42 |
| *IL32* | 16 | 16896 | 2517 | 225 | 265 | 184 |
| *JUN* | 1 | 3257 | 2286 | 0 | 0 | 0 |
| *JUNB* | 19 | 1830 | 2634 | 0 | 0 | 0 |
| *JUND* | 19 | 1929 | 2723 | 0 | 0 | 0 |
| *KLF6* | 10 | 9286 | 3627 | 0 | 0 | 0 |
| *NKG7* | 19 | 1096 | 3400 | 2 | 0 | 0 |
| *SOCS3* | 17 | 3300 | 3603 | 0 | 0 | 0 |

Supplementary Table S3: External validation of *IL32* eSNPs identified by BPHurdle in CD4+ Th1 cells using publicly available eQTL resources, including GTEx, eQTLGen, and DICE.

| rsID | SNP | Cell Type | GTEx | eQTLGen | DICE | Confirmed |
|---|---|---|---|---|---|---|
| rs11554091 | chr16_3508381 | CD4+ Th1 | No | No | No | No |
| rs9972684 | chr16_3710335 | CD4+ Th1 | No | No | No | No |
| rs2238441 | chr16_3985406 | CD4+ Th1 | No | No | No | No |
| rs190438582 | chr16_2137278 | CD4+ Th1 | No | No | No | No |
| rs27699 | chr16_2206325 | CD4+ Th1 | No | No | No | No |
| rs61204087 | chr16_2368178 | CD4+ Th1 | No | No | No | No |
| rs78764216 | chr16_2672165 | CD4+ Th1 | No | No | No | No |
| rs76634649 | chr16_2742509 | CD4+ Th1 | No | No | No | No |
| rs62032288 | chr16_2877770 | CD4+ Th1 | No | No | No | No |
| rs113617058 | chr16_2901858 | CD4+ Th1 | No | Yes | No | Yes |
| rs8052002 | chr16_3020787 | CD4+ Th1 | Yes | Yes | No | Yes |
| rs4786377 | chr16_3075426 | CD4+ Th1 | Yes | Yes | No | Yes |
| rs2717685 | chr16_3097673 | CD4+ Th1 | No | Yes | No | Yes |
| rs2252009 | chr16_3164441 | CD4+ Th1 | No | No | No | No |
| rs33980223 | chr16_3328146 | CD4+ Th1 | No | No | No | No |
| rs56093151 | chr16_3577016 | CD4+ Th1 | No | No | No | No |
| rs3751842 | chr16_3664610 | CD4+ Th1 | No | No | No | No |
| rs2072379 | chr16_3688886 | CD4+ Th1 | No | No | No | No |
| rs11646233 | chr16_3947223 | CD4+ Th1 | No | No | No | No |
| rs2238427 | chr16_3953762 | CD4+ Th1 | No | No | No | No |
| rs2230742 | chr16_3966675 | CD4+ Th1 | No | No | No | No |
| rs2230739 | chr16_3983435 | CD4+ Th1 | No | No | No | No |

Supplementary Table S4: External validation of *IL32* eSNPs identified by BPHurdle in CD4+ Th2 cells using publicly available eQTL resources, including GTEx, eQTLGen, and DICE.

| rsID | SNP | Cell Type | GTEx | eQTLGen | DICE | Confirmed |
|---|---|---|---|---|---|---|
| rs26862 | chr16_2207104 | CD4+ Th2 | No | No | No | No |
| rs6500612 | chr16_2797516 | CD4+ Th2 | No | No | No | No |
| rs8052002 | chr16_3020787 | CD4+ Th2 | Yes | Yes | No | Yes |
| rs2245000 | chr16_3025998 | CD4+ Th2 | Yes | Yes | No | Yes |
| rs2079244 | chr16_3028543 | CD4+ Th2 | Yes | Yes | No | Yes |
| rs2013921 | chr16_3054845 | CD4+ Th2 | No | Yes | Yes | Yes |
| rs59485676 | chr16_3064627 | CD4+ Th2 | No | Yes | No | Yes |
| rs12934561 | chr16_3068864 | CD4+ Th2 | Yes | Yes | No | Yes |
| rs117895714 | chr16_3071620 | CD4+ Th2 | No | No | No | No |
| rs11643422 | chr16_3159667 | CD4+ Th2 | No | Yes | No | Yes |
| rs224225 | chr16_3254762 | CD4+ Th2 | No | No | No | No |
| rs7206826 | chr16_3466162 | CD4+ Th2 | No | No | No | No |
| rs11554091 | chr16_3508381 | CD4+ Th2 | No | No | No | No |
| rs9925417 | chr16_3529803 | CD4+ Th2 | No | No | No | No |
| rs11646287 | chr16_3669343 | CD4+ Th2 | No | No | No | No |
| rs9972684 | chr16_3710335 | CD4+ Th2 | No | No | No | No |
| rs12446940 | chr16_3912619 | CD4+ Th2 | No | No | No | No |
| rs7196217 | chr16_3940829 | CD4+ Th2 | No | No | No | No |
| rs2238427 | chr16_3953762 | CD4+ Th2 | No | No | No | No |
| rs17794327 | chr16_3981932 | CD4+ Th2 | No | No | No | No |
| rs2601817 | chr16_4027137 | CD4+ Th2 | No | No | No | No |

Supplementary Table S5: External validation of *IL32* eSNPs identified by BPHurdle in CD4+ Th17 cells using publicly available eQTL resources, including GTEx, eQTLGen, and DICE.

| rsID | SNP | Cell Type | GTEx | eQTLGen | DICE | Confirmed |
|---|---|---|---|---|---|---|
| rs28372698 | chr16_3065110 | CD4+ Th17 | Yes | No | No | Yes |
| rs2717685 | chr16_3097673 | CD4+ Th17 | No | Yes | No | Yes |
| rs12934426 | chr16_3159481 | CD4+ Th17 | No | No | No | No |
| rs2252009 | chr16_3164441 | CD4+ Th17 | No | No | No | No |
| rs33980223 | chr16_3328146 | CD4+ Th17 | No | No | No | No |
| rs11554091 | chr16_3508381 | CD4+ Th17 | No | No | No | No |
| rs9972684 | chr16_3710335 | CD4+ Th17 | No | No | No | No |
| rs2230742 | chr16_3966675 | CD4+ Th17 | No | No | No | No |
| rs2601817 | chr16_4027137 | CD4+ Th17 | No | No | No | No |
